# ADHD Characteristics Are Linked to Divergent Risk-Taking Behaviors

**DOI:** 10.1177/10870547261432427

**Published:** 2026-03-31

**Authors:** Anselm B. M. Fuermaier, Hui Dong, Yehuda Pollak, Barbara R. Braams, Tycho J. Dekkers

**Affiliations:** 1 University of Groningen, The Netherlands; 2 The Hebrew University of Jerusalem, Israel; 3 Vrije Universiteit Amsterdam, The Netherlands; 4 Accare Child Study Center, Groningen, The Netherlands; 5 University Medical Center Groningen, The Netherlands; 6 Levvel, Academic Center for Child and Adolescent Psychiatry, Amsterdam, The Netherlands

**Keywords:** adult ADHD characteristics, risk-taking, negative risk-taking, positive risk-taking, prosocial risk-taking

## Abstract

**Objectives:**

Decades of research on individuals with ADHD across the lifespan have consistently demonstrated an increased tendency for negative risk-taking. However, the potential for positive forms of risk-taking has been largely overlooked. Very recently, Braams et al. were the first to report an increased likelihood of prosocial risk-taking, a subtype of positive risk-taking, among adolescents with ADHD. Building on their findings, the present preregistered study investigates self-reported likelihoods of engaging in negative, positive, and prosocial risk-taking and examines their associations with ADHD characteristics, as well as internalizing symptoms as comparison measures.

**Methods:**

This correlational study analyzed survey data from 611 participants drawn from the Dutch general population. Participants rated their likelihood of engaging in negative, positive, and prosocial risk-taking behaviors and completed standardized questionnaires assessing ADHD characteristics as well as internalizing symptoms of depression, anxiety, and stress. Correlation analyses, regression models, and categorical analyses were used to examine the unique and combined contributions of ADHD characteristics and internalizing symptoms to different forms of hypothetical risk-taking behavior.

**Results:**

ADHD characteristics were robustly associated with negative risk-taking likelihood. Associations with positive and prosocial risk-taking were present but weaker and less consistent across analytical methods. Hierarchical regression analyses indicated that ADHD traits accounted for a substantial portion of the variance in risk-taking behaviors, while internalizing symptoms also contributed, though to a lesser extent. The likelihood of engaging in risk-taking behavior decreased with increasing age.

**Conclusion:**

Our findings support the emerging view that ADHD characteristics may be linked not only to negative but also to positive and prosocial forms of risk-taking. However, effects on positive and prosocial risk taking are more complex and warrant further research, particularly with clinical samples and psychometrically refined instruments. Our findings may foster a more nuanced understanding of ADHD and could have important implications for self-concept development and clinical care in individuals with ADHD.

## Introduction

The vast majority of risk-taking research focuses on negative and undesirable outcomes, and thereby often neglects a potential positive side of risk-taking behavior. This is surprising, because when applying neuroeconomic models of decision-making, risk is defined by three key components, that is (1) variability in the likelihood of outcomes, (2) uncertainty about the outcome, and (3) the potential for both costs *and benefits* (Crone et al., 2016; Duell & Steinberg, 2019; Holton, 2004). Consequently, risk-taking behavior, in general, is not limited to negative or undesirable actions and outcomes but can also have an advantageous side. Positive risk-taking refers to behaviors that, while involving some level of uncertainty, have the potential to yield personal benefits. These behaviors are often linked to extraversion and sensation seeking and may include giving a public presentation, trying out for a sports team, initiating new social connections, traveling to unfamiliar places, enrolling in a challenging course, or applying for an attractive job opportunity. A third category of risk-taking, related to positive risk-taking, is prosocial risk-taking, in which the risky behavior is primarily intended to benefit others rather than oneself (Do et al., 2017). Examples include standing up for someone being bullied, missing work to participate in climate change protests, informing someone that they are being talked about behind their back, or intervening in a physical dispute. While prosocial risk-taking is also associated with sensation seeking, it additionally correlates with empathy and prosocial motivation (Armstrong-Carter et al., 2021; Braams et al., 2025).

Research on various forms of risk-taking is particularly interesting in populations which are commonly associated with taking more risks than others. ADHD is characterized by age-inappropriate levels of inattention, hyperactivity, and impulsivity. While ADHD is typically diagnosed in childhood, approximately half of the affected individuals continue to meet diagnostic criteria into adulthood, with a global prevalence of persistent adult ADHD of about 2.58% (Song et al., 2021). Core characteristics of ADHD, particularly impulsivity, are strongly linked to various forms of risk-taking behavior (Pollak et al., 2019; Romer, 2010). A large extent of research has demonstrated associations between adult ADHD and risk-taking behaviors that often lead to negative outcomes. These include increased alcohol and drug use (Molina & Pelham, 2014), aggression and criminal behavior (Dayan et al., 2023; Mohr-Jensen et al., 2019; Pratt et al., 2002), reckless and dangerous driving (Barkley & Cox, 2007; Fuermaier et al., 2017), risky financial decisions and gambling (Bangma et al., 2019; Breyer et al., 2009; Theule et al., 2016), food-related risk-taking (Hershko et al., 2018; Kaisari et al., 2017), as well as unprotected sex and unplanned pregnancies (Flory et al., 2006; Ostergaard et al., 2017). However, to date, an advantageous side of risk-taking is an aspect that has received relatively little attention in ADHD research.

Whereas the clinical assessment and treatment of adults with ADHD, plausibly, focuses on identifying problems and designing and implementing interventions, recognizing potential strengths associated with ADHD could play a crucial role in fostering self-concept, promoting acceptance of problems, and supporting coping strategies. Additionally, this perspective may help clinicians refine psychoeducation and compensation training for individuals with ADHD (Schippers et al., 2022). In a recent cross-sectional study on adolescents with ADHD, Braams et al. (2025) found that adolescents with ADHD reported a higher likelihood of engaging in prosocial risk-taking compared to controls. However, contrary to the authors' expectations, no significant differences were found in negative or positive risk-taking between the adolescents with and without ADHD.

The present study is the first to examine the relationship between ADHD characteristics and both positive and prosocial risk-taking behavior in adults. In our study, we adopt a dimensional approach to ADHD, that positions individuals on a continuum of symptomatology rather than categorizing them into discrete diagnostic groups (for discussion on the dimensional approach, see Hudziak et al., 2007; for application, see Mohamed et al., 2015). This approach acknowledges that ADHD characteristics^1^ can manifest in various clinical conditions and also at subclinical levels (Sibley, 2021). For the purpose of this study, we recruited a Dutch community sample of 888 individuals who completed questionnaires assessing negative, positive, and prosocial risk-taking, as well as self-reported characteristics of ADHD, depression, anxiety, and stress. Given that positive and negative risk-taking are linked both conceptually and empirically (Braams et al., 2025; Do et al., 2017), we hypothesized that ADHD characteristics in adults were associated with higher levels of negative, positive, and prosocial risk-taking. Further, we expect that ADHD characteristics are associated with risk-taking above and beyond general indications of mental health, for example, internalizing symptoms of depression, anxiety, and stress. Because we expected risk-taking to be specifically associated with ADHD characteristics and not generally with indications of mental health, we did not expect internalizing symptoms of depression, anxiety, or stress to be significantly associated with any form of risk-taking. Additionally, we categorized participants into an elevated ADHD characteristics group (endorsing at least five symptoms of inattention and/or hyperactivity/impulsivity) and a comparison group not meeting these criteria (i.e., endorsing none or up to four symptoms in each of the symptom domains). Consistent with our correlational hypotheses, we expected that individuals with elevated ADHD characteristics exhibit higher levels of negative, positive, and prosocial risk-taking compared to those with no elevated ADHD characteristics.

## Methods

We preregistered our study protocol at AsPredicted (https://aspredicted.org/rk67-kkc3.pdf). We followed the pre-registered plan and reported when we deviated from the protocol.

### Participants

Participants were recruited from PanelInzicht, a Dutch online platform which invites individuals to register and take part in online studies for a financial reward. The questionnaire was designed and administered with the survey tool Qualtrics. We invited adults aged 18 to 65 years, with matching for age and gender, from the Dutch community between March 11, 2025 and April 7, 2025. A total of 888 participants completed the survey and were considered for inclusion in this study. Online surveys are vulnerable to careless responding where participants may respond randomly, inattentively, or without sufficient engagement (Ward & Meade, 2022). For this reason, we employed a multi-method approach to identify and exclude low-quality responses (see statistical analysis, and Table 1). In addition to attention check items and survey completion time as indicated at preregistration, we decided to make use of further validity indices (see Table 1) to improve data quality. We excluded 277 participants with likely careless responses and retained a sample of 611 participants for the final analysis, which exceeds the aimed sample size of *N* = 400 as indicated at preregistration.

**Table 1.** Exclusion Criteria for Careless Responding.

Index class	Index	Classification criterion	Number of participants excluded
Validity items	Attention checks	Item 30 ≠ 2; Item 13 ≠ 4; Item 10 ≠ 3	102
Invariability	IRV	*Z*-score <−1.64; *Z*-score > 1.64	72
Consistency	Long-string	*Z*-score > 2	151
Time	Completion time	<2 s/item (109 items = 218 s)	51
Manual inspection	Repeated responses	Double participation to survey	6
	Random responses	Nonsense entries in open question	3
Total careless responses			277

*Note.* Total careless responses is no sum score of the criteria above as it takes into consideration that some participants are flagged by more than one criterion. IRV = intra-individual Response Variability.

A subgroup of individuals was categorized as endorsing elevated ADHD characteristics based on their self-reported ADHD symptoms. The elevated ADHD characteristics group (*n* = 114) was comprised of individuals endorsing ≥5 symptoms (item score >2) in either inattention and/or hyperactivity/impulsivity domains (corresponding to DSM-5 diagnostic thresholds for current symptom experience). The comparison group (*n* = 114) was sampled from all participants who do not endorse elevated ADHD characteristics (*n* = 497), selected through 1:1 nearest-neighbor matching based on age and gender. Endorsement of ADHD characteristics was assessed using a common ADHD screening instrument, which is not equivalent to a comprehensive, multi-informant diagnostic evaluation that includes information on childhood behavior and functional impairment. Therefore, the results of this screening should not be interpreted as evidence of a stable ADHD trait, but rather as reflecting current experiences of ADHD-related characteristics. ADHD self-report screening tools are known to have high sensitivity (meaning they effectively identify most individuals with ADHD as having ADHD) but low specificity (indicating that they too often incorrectly identify individuals without ADHD as having ADHD). Most importantly for this context, when estimated on realistic base rates of ADHD, their positive predictive values are generally low (for those who tested positive, the likelihood of truly having ADHD is not high; see Harrison & Edwards, 2023). The characteristics of participants can be found in Table 2. Group differences in age were tested by a Wilcoxon rank-sum test between the groups. To assess group differences in categorical variables (i.e., gender and education), Pearson’s chi-square tests were used. Effect sizes were reported using Cohen’s *d* for continuous variables and Cramer’s *V* for categorical variables (Cramér, 1999).

**Table 2.** Group Characteristics (*M* ± *SD*) and Comparisons Between the Elevated ADHD Characteristics and Comparison Group.

	Total sample (*N* = 611)	Elevated ADHD characteristics^a^ (*n* = 114)	Comparison group^b^ (*n* = 114)	*p*	Cohen’s *d*/Cramér’s *V*
Gender identity^c^ (female/male/non-binary)	342/263/5	62/50/2	67/46/1	.701	0.03
Age (in years)	43.85 ± 15.33	34.84 ± 14.25	35.58 ± 13.83	.633	0.04
Education (1/2/3/4/5/6/7)^d^	1/13/109/54/35/151/74	0/0/15/7/8/30/22	1/1/14/12/10/36/17	.692	0.16
Risk taking behavior					
Negative risk taking	61.08 ± 13.87	68.79 ± 12.97	62.38 ± 13.05	<.001***	0.49
Positive risk taking	38.97 ± 10.50	41.80 ± 10.16	39.75 ± 10.88	.164	0.19
Prosocial risk taking	42.05 ± 7.58	44.41 ± 6.47	42.96 ± 7.86	.158	0.20
Symptom scores					
ADHD	17.10 ± 10.55	33.68 ± 7.80	12.98 ± 6.46	<.001***	2.89
Depression	10.94 ± 4.19	14.45 ± 4.74	9.31 ± 3.06	<.001***	1.29
Anxiety	10.32 ± 3.44	13.58 ± 3.99	9.25 ± 2.58	<.001***	1.29
Stress	12.15 ± 4.09	16.68 ± 4.06	10.07 ± 2.76	<.001***	1.90

a Elevated ADHD characteristics was operationally defined as endorsing ≥ 5 symptoms of inattention and/or hyperactivity/impulsivity.

b Comparison group was sampled from participants who did not meet the criteria for elevated ADHD characteristics.

c Gender was not reported from one individual of the total sample. Non-binary participants were not considered in the group comparison. Group differences in gender (female vs. male) were assessed using Pearson’s chi-square test.

d Education (1/2/3/4/5/6/7) = Ordinal presentation of Dutch education level, ranging from primary school without educational training until scientific education in BSc/MSc. Education level was not reported in 174 cases (32 in the elevated ADHD characteristics group and 23 in the comparison group). Fisher’s exact test was applied because some cells had small expected counts.

Significant differences between groups are indicated by *p < .05, **p < .01, and ***p < .001.

### Measures

All items of the negative, positive, and prosocial risk-taking scale can be found in the Supplemental Material.

#### Negative Risk-Taking

We assessed the likelihood of engaging in negative risk-taking behavior with a selection of 20 items of the DOSPERT (i.e., the domain-specific risk-taking scale, e.g., “Drinking heavily at a social function,” Blais & Weber, 2006) with forced response entry. Each item can be allocated to one of five subscales, indicating ethical, financial, health/safety, recreational, and social aspects of negative risk-taking. Each item represents a statement on negative risk-taking behavior, and participants are asked to indicate how likely they would engage in this behavior on a 7-point Likert scale ranging from 1 (“extremely unlikely”) to 7 (“extremely likely”). We calculate a total negative risk-taking sum score. The negative risk-taking scale had an internal consistency of Cronbach’s α = .78 on the final sample of this study (*N* = 611).

#### Positive Risk-Taking

Hypothetical engagement in positive risk-taking behavior was measured with an adaptation of the positive risk-taking questionnaire (posRT). The posRT was based on the DOSPERT (Blais & Weber, 2006) and adapted by Braams et al. (2025; e.g., “Giving a presentation to a full room”). We selected 10 items (forced response entry) that appeared most relevant to restrict the total survey length, and interspersed the items in the negative risk-taking questionnaire and applied the same Likert scale, which was the likelihood of engaging in this behavior ranging from 1 (“extremely unlikely”) to 7 (“extremely likely”). We calculated a sum score for an indication of positive risk-taking. The positive risk-taking scale had an internal consistency of Cronbach’s α = .78 on the final sample of this study (*N* = 611).

#### Prosocial Risk-Taking

We assessed the hypothetical engagement in engaging in prosocial risk-taking behavior with the Prosocial Risk-Taking (PSRT) questionnaire. The PSRT was developed by Braams et al. (2025, e.g., “Making a test for a friend”) and was based on the DOSPERT (Blais & Weber, 2006). We selected 10 items (forced response entry) of the PSRT that appeared most relevant in order to restrict the total survey length, and interspersed the items in the posRT and negative risk-taking questionnaire, and applied the same Likert Scale, which was the likelihood of engaging in this behavior ranging from 1 (“extremely unlikely”) to 7 (“extremely likely”). The prosocial risk-taking scale had an internal consistency of Cronbach’s α = .66 on the final sample of this study (*N* = 611).

#### ADHD Characteristics

ADHD characteristics were assessed with the Dutch version of the adult ADHD Self-Report Scale (ASRS; Kessler et al., 2005; Kooij & Buitelaar, 1997; Kooij et al., 2008) with forced response entry. The ASRS contains 23 items (e.g., “I make careless mistakes at work”) that are answered on a 4-point scale ranging from 0 (“rarely or never”) to 3 (“very often”) over the past 6 months. Each DSM-5 ADHD symptom is reflected by one item, except for five symptoms which are reflected by two items. A sum score of ADHD characteristics is calculated for which double DSM-5 items are averaged. Further, we determined “elevated ADHD characteristics” if an individual endorses at least five ADHD DSM-5 symptoms (score >2) for inattention and/or hyperactivity/impulsivity.

#### Depression, Anxiety, and Stress

The Depression Anxiety Stress Scale-21 (DASS-21) was applied as a self-report questionnaire for internalizing symptoms of depression (e.g., “I found it difficult to take the initiative to do something”), anxiety (e.g., “I felt I was about to panic”), and stress (e.g., “I found it difficult to calm myself down”) over the past 6 months (De Beurs et al., 2001; Lovibond & Lovibond, 1995). It consists of 21 items, with seven items per subscale, rated on a 4-point Likert scale ranging from 0 (“Did not apply to me at all”) to 3 (“Applied to me very much or most of the time”). We calculated sum scores for each subscale. Each sum score is doubled to match the original version of 42 items.

### Procedure

The study protocol was approved by the ethics committee psychology (ECP) of the University of Groningen (approval number PSY-2425-S-0196; approval date: 26.2.2025). The survey started with the risk-taking questionnaire, followed by the ASRS and DASS-21, and took about 10 min to complete. An additional small set of 11 items were part of the survey but not part of and presented in this study.

### Statistical Analysis

#### Careless Responding and Participant Exclusion

Three *attention check* items were interspersed in the survey to proactively screen for inattentive or disengaged responding. These items required participants to select a predetermined correct response (e.g., “Please select the response ‘*extremely unlikely*’”), serving as a direct test of whether participants were reading the items carefully. These methods are typically employed in the midst of lengthy, single-administration surveys (Meade & Craig, 2012). Participants who failed to respond correctly to any of the three embedded attention check items were excluded from further analysis (*n* = 102).

The in-person variability across items was measured by the *Intra-individual Response Variability* (*IRV*; Dunn et al., 2018). Low IRV could potentially indicate careless responding, however, a higher IRV may also be considered as random responding (Dunn et al., 2018). Given that IRV performs optimally when computed across 25 to 150 items (Dunn et al., 2018), we combined all items from the three administered questionnaires (Risk-Taking, 40 items; ASRS, 23 items; and DASS-21, 21 items), resulting in an 84-item composite suitable for IRV calculation. Prior to analysis, we standardized responses within each questionnaire separately to a common metric (*z*-scores) to account for differences in rating scales (i.e., 4-point Likert of DASS vs. 7-point Likert Risk-Taking). This ensured comparability across measures while preserving within-person response variability. Following Huang et al. (2012), participants whose IRV values fell above or below 1.64 standard deviations from the sample mean (i.e., *z* < −1.64 or *z* > 1.64) were excluded (*n* = 72).

The *longstring index* (Johnson, 2005) is the longest consecutive number of the same response in a questionnaire which may indicate a rushed and careless response style. Like the IRV index, the longstring index is most meaningful in the context of multi-construct questionnaires in which the order of the items is randomized across the questionnaire. For example, in the response sequence “2, 2, 1, 5, 5, 5, 5, 1,” the long-string index would be 4. To avoid obscuring response patterns due to differences in rating scales, we computed the longstring index separately for each questionnaire. This ensures that consistent extreme responding (e.g., a careless responder may always choose the highest option “7” on one scale and the highest option “4” on another) is accurately captured within each scale format. Following previous studies (Charpentier et al., 2024; Wu et al., 2024, 2025), we excluded participants (*n* = 151) whose longstring index exceeded two standard deviations from the mean (i.e., *z*-score >2).

We checked the *survey completion time* of participants and excluded data of participants who responded faster than on average 2 s per item (Huang et al., 2012). Fifty-one individuals were excluded from the analyses based on this criterion.

Further, a *manual inspection* of data lead to the exclusion of nine responses based on clear evidence of random responding in open questions (e.g., nonsense entries in a final comment box) and/or repeated responding.

#### Hypothesis Testing

Our analytical strategy proceeded in three stages: First, Spearman’s rank-order correlations were computed on the total sample (*N* = 611) to examine bivariate associations between symptom dimensions (ADHD, depression, anxiety, and stress) and risk-taking behaviors. Second, hierarchical linear regressions were conducted on the total sample to assess the distinct effect of ADHD and incremental effect of internalizing symptoms (depression, anxiety, stress) on each of the three risk-taking domains (negative, positive, and prosocial). Finally, and third, we tested for group differences between the elevated ADHD characteristics group and the matched comparison group across three risk-taking domains (negative, positive, and prosocial) and four symptom dimensions (ADHD, depression, anxiety, stress). This analysis adds relevance by categorizing individuals, albeit based on self-reports, into groups corresponding to the DSM-5 diagnostic threshold for current ADHD symptom experiences (i.e., endorsing ≥5 characteristics in either or both symptom domains). Group comparisons of this kind can provide a basis for expectations and hypotheses in subsequent clinical studies.

Group comparisons on continuous variables were conducted using nonparametric statistics (Wilcoxon rank-sum tests) due to violations of assumptions of normality and homogeneity of variances. Cohen’s *d* effect size indicates the magnitude of the findings. According to the interpreting guidelines, a Cohen’s *d* of <0.2 indicates a negligible effect, 0.2 to 0.49 a small effect, 0.5 to 0.79 a medium effect, and ≥ 0.8 a large effect (Cohen, 2013).

Our analysis plan was preregistered at AsPredicted (https://aspredicted.org/rk67-kkc3.pdf). All analyses were performed in R (v4.4.1; R Core Team, 2021). We used the “careless” R package (Yentes & Wilhelm, 2021) to compute indices for careless responses. The comparison group to the group with elevated ADHD characteristics was identified via the MatchIt package (version 4.5.5, Ho et al., 2018) in R, ensuring demographic comparability.

## Results

### Exploratory Analyses

As indicated at preregistration, we performed additional exploratory analyses on scale and item levels. First, we calculated the correlation between risk-taking and age, that is for negative risk-taking (Spearman’s *r* = −.17; *p* < .001), positive risk-taking (*r* = −.15; *p* < .001), and prosocial risk-taking (Spearman’s *r* = −.28; *p* < .001). Because of significant age effects, we added age as a covariate to our models of hypothesis testing (i.e., correlation analysis and hierarchical linear regressions) and presented additional data in Supplemental Table S1 on age-stratified samples showing group comparisons. While controlling for age was not indicated in age-matched group comparisons, we tested in exploratory analysis whether age moderated group effects on various risk taking dimensions.

Second, we calculated Spearman correlations between different types of risk-taking, that is, between negative and positive risk-taking (*r* = .54, *p* < .001), negative and prosocial risk-taking (*r* = .45, *p* < .001), as well as positive and prosocial risk-taking (*r* = .51, *p* < .001).

Finally, and third, we performed exploratory single item analyses on the prosocial risk-taking scale, because reliability analysis of this scale (10 items) revealed only modest internal consistency (Cronbach’s alpha = .66). Item-level diagnostics identified Q30 (“Stay away from work to participate in a climate protest”) as an underperforming item with an item-total correlation of .15, which is substantially below the conventional threshold of .30. Removing Q30 increased the alpha to no further than .68. Sequential removal of other weakly correlated items marginally improve alpha to .69, which indicates scale reliability cannot be improved substantially by item deletion.

### Correlation Analysis

**Figure 1.**
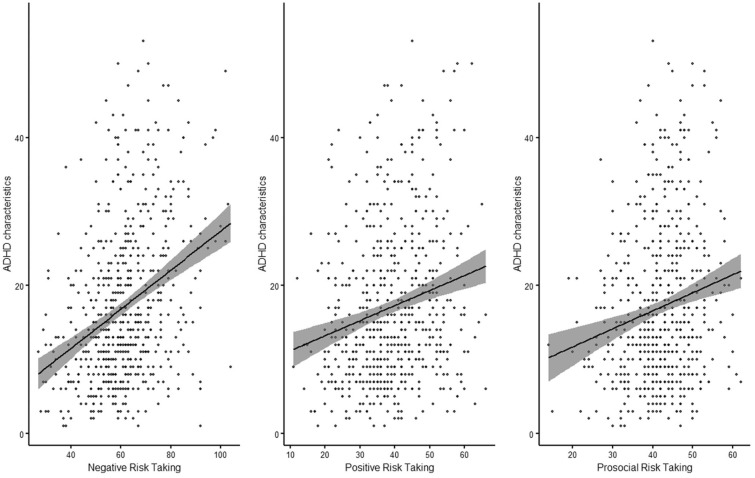
Visualization of the relationships between different forms of risk-taking behavior (negative, positive, and prosocial risk-taking) and ADHD characteristics (*N* = 611). Each panel shows a linear regression line with shaded 95% confidence intervals.

Bivariate analyses (Table 3, controlling for age) revealed that ADHD characteristics positively correlated with all risk-taking types, with the strongest correlation observed for negative risk-taking (*r* = .28), followed by positive (*r* = .16) and prosocial risk-taking (*r* = .12). Depression was significantly positively related to negative risk-taking (*r* = .13) and prosocial risk-taking (*r* = .08), but not to positive risk-taking (*r* = −.04). Anxiety was significantly associated with negative (*r* = .16) and prosocial (*r* = .16) risk taking. Stress also showed significant positive correlations with negative (*r* = .21), positive (*r* = .08), and prosocial risk-taking behavior (*r* = .13). The relationships between ADHD characteristics and the three risk-taking dimensions are visualized in Figure 1 (uncontrolled for age).

**Table 3.** Partial Spearman Correlations (Controlling for Age) Between Different Forms of Risk-taking Behaviors and ADHD Characteristics, Depression, Anxiety, and Stress.

Risk taking behavior	Symptom scores			
	ADHD	Depression	Anxiety	Stress
Negative risk taking	0.28***	0.13***	0.16***	0.21***
Positive risk taking	0.16***	−0.04 (ns)	0.05 (ns)	0.08**
Prosocial risk taking	0.12**	0.08*	0.16***	0.13***

*Note.* “ns” indicates non-significant correlations (*p* > .05).

* *p* < .05. **p < .01. ***p < .001.

### Regression Analysis

To examine the distinct and incremental effects of ADHD characteristics and internalizing symptoms (depression, anxiety, and stress) on different dimensions of risk-taking, hierarchical regression analyses were conducted separately for positive, prosocial, and negative risk-taking, controlling for age (Table 4). In step 1, only ADHD characteristics and age were included, whereas in step 2, depression, anxiety, and stress were added to assess their incremental predictive power. The Variance Inflation Factors for all predictors (ADHD = 2.17, Depression = 2.48, Anxiety = 2.20, Stress = 3.61, and age = 1.16) are below the commonly used threshold of 5, indicating that multicollinearity was no major concern in this model. For negative risk-taking, both ADHD characteristics and age were a strong positive predictor in step 1, and this association remained significant in step 2. However, none of the internalizing symptoms significantly predicted negative risk-taking in step 2, Δ*R*² = .003, *F* = 0.63, *p* = .594. For positive risk-taking, ADHD characteristics and age were a significant positive predictor in step 1, and remained significant in step 2. Depression emerged as a significant negative predictor while anxiety and stress were non-significant, Δ*R*² = .019, *F* = 4.18, *p* = .006. For prosocial risk-taking, ADHD characteristics and age were initially a significant predictor, but the effect became non-significant after adding internalizing symptoms in step 2. Anxiety emerged as a significant positive predictor, and depression showed a marginal negative trend with an improved model fit, Δ*R*² = .016, *F* = 3.31, *p* = .020.

**Table 4.** Hierarchical Linear Regression Models: Stepwise Inclusion of ADHD and Internalizing Symptoms Predicting Three Types of Risk-taking Behavior, Controlling for Age.

Outcome variable	Step	Predictor	Estimate	*SE*	*t*	*p*	*R*²	Δ*R*²	*F* for Δ*R*²
Negative risk taking	1	ADHD	0.38	0.05	7.28	<.001***	.14		
		Age	−0.14	0.04	−3.99	<.001***			
	2	ADHD	0.32	0.07	4.34	<.001***	.15	.003	0.633 (ns)
		Age	−0.14	0.04	−3.94	<.001***			
		Depression	0.03	0.20	0.14	.888			
		Anxiety	0.08	0.22	0.36	.718			
		Stress	0.17	0.24	0.71	.476			
Positive risk taking	1	ADHD	0.17	0.04	3.99	<.001***	.05		
		Age	−0.07	0.03	−2.38	.018*			
	2	ADHD	0.20	0.06	3.54	<.001***	.07	.019	4.175**
		Age	−0.06	0.03	−2.04	.042*			
		Depression	−0.50	0.16	−3.23	.001**			
		Anxiety	0.24	0.18	1.33	.184			
		Stress	0.09	0.19	0.48	.629			
Prosocial risk taking	1	ADHD	0.10	0.03	3.36	<.001***	.04		
		Age	−0.05	0.02	−2.24	.025*			
	2	ADHD	0.04	0.04	0.88	.380	.05	.016	3.307*
		Age	−0.04	0.02	−1.91	.057			
		Depression	−0.18	0.11	−1.55	.121			
		Anxiety	0.32	0.13	2.49	.013*			
		Stress	0.16	0.14	1.14	.255			

*Note.* ns = non-significant.

* *p* < .1. **p < .05. ***p < .01.

### Group Comparisons

**Figure 2.**
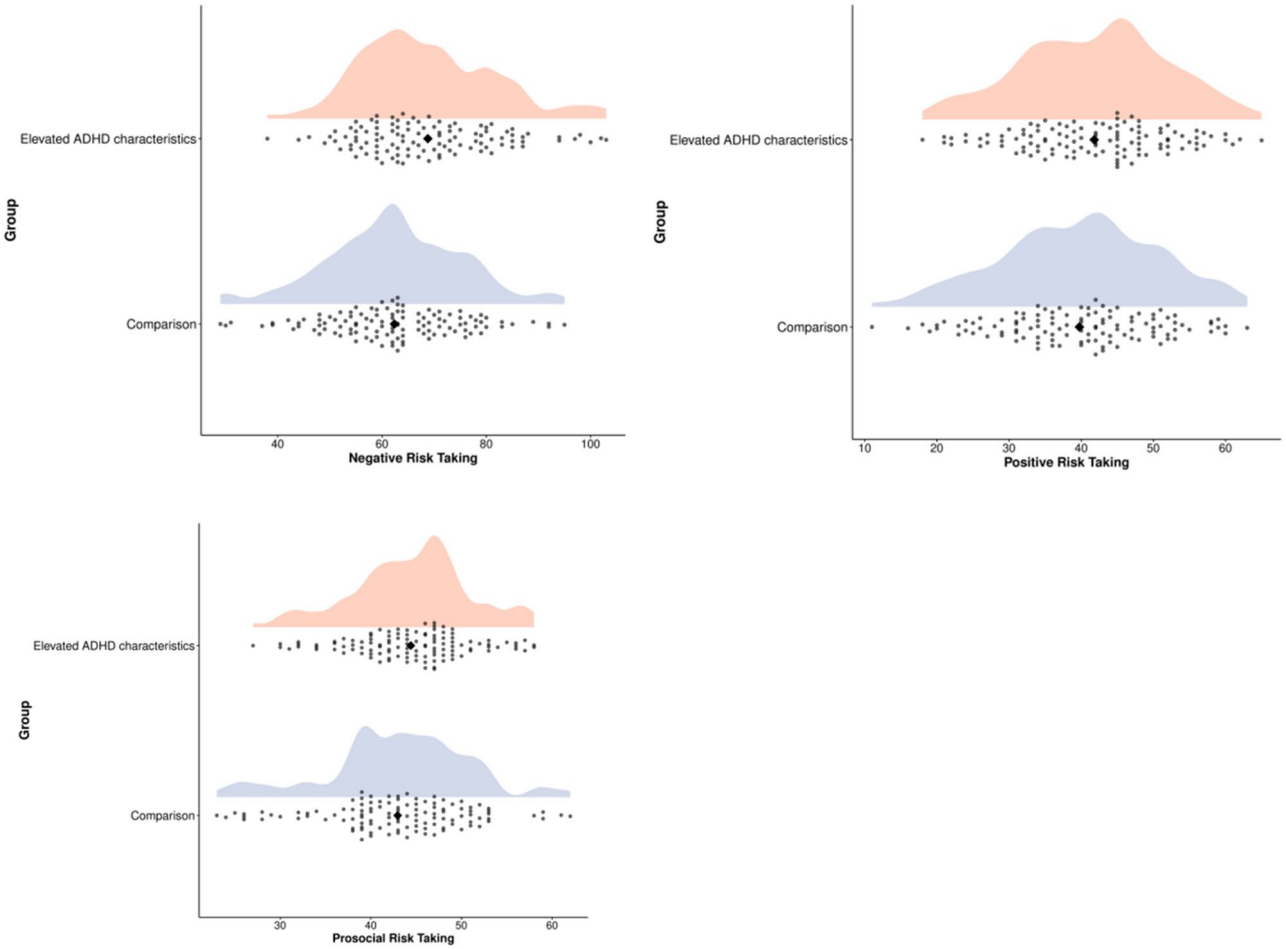
Distribution of the three different forms risk-taking behavior in the elevated ADHD characteristics group (*n* = 114) and comparison group (*n* = 114). The orange density clouds represent the elevated ADHD characteristics group, while the blue density clouds represent the comparison group. Diamonds indicate the mean value for each group.

As presented in Table 2, the elevated ADHD characteristics group and the comparison group did not differ significantly in gender, age, or education, ensuring demographic comparability. However, as expected from group assignment based on ADHD symptom endorsement, the group with elevated ADHD characteristics exhibited markedly higher symptom severity across all domains, with large effect sizes (ADHD: *d* = 2.89; depression: *d* = 1.29; anxiety: *d* = 1.29; stress: *d* = 1.90; all *p* < .001). In contrast, risk-taking scores diverged significantly only for negative risk-taking (higher risk-taking shown by the group with elevated ADHD characteristics: *d* = 0.49), but not positive (*d* = 0.19) or prosocial (*d* = 0.20) risk-taking (Figure 2 for a visualization of the effects, uncontrolled for age). Because age seemed to be related to various forms of risk taking as shown in the exploratory analysis, we additionally repeated the group comparisons in age-stratified subsamples to examine whether age moderated the differences between the elevated ADHD characteristics group and the comparison group and found no such effect (see Supplemental Table S1).

## Discussion

In this preregistered cross-sectional study using a large community sample from the Netherlands, we hypothesized that negative, positive, and prosocial forms of risk-taking would be associated with ADHD characteristics, but not with symptoms of depression, anxiety, or stress. We expected this pattern to emerge across correlational analyses, regression models, and group comparisons.

The results only partially confirmed our expectations. Correlational analyses indicated that ADHD characteristics were indeed associated with negative, positive, and prosocial risk-taking, with the strongest associations observed for negative risk-taking. In contrast, the associations between all forms of risk-taking and symptoms of depression, anxiety, and stress, were generally weaker and less consistent, although several reached statistical significance, particularly for negative and prosocial risk-taking. Hierarchical multiple regression analyses supported the patterns observed in the age-controlled correlations. ADHD characteristics were significantly associated with all types of risk-taking, explaining up to 14% of the variance in negative risk-taking. After adding internalizing symptoms in Step 2, ADHD characteristics remained a significant predictor of negative and positive risk-taking, whereas the association with prosocial risk-taking became non-significant. The inclusion of internalizing symptoms in the models led to only modest increases in explained variance. Notably, internalizing symptoms did not significantly predict negative risk-taking, whereas depression emerged as a predictor of positive risk-taking and anxiety as a predictor of prosocial risk-taking. However, group comparisons between individuals with elevated ADHD characteristics and a matched control group did not yield consistent results. While the elevated ADHD characteristics group reported a significantly higher likelihood of engaging in negative risk-taking behavior, differences in positive and prosocial risk-taking were negligible. The correlation between ADHD characteristics and negative risk-taking appears sufficient to translate into significant group-level differences. In contrast, the small correlations with positive and prosocial risk-taking may not be strong enough to generate significant effects when comparing individuals at the extremes of the ADHD characteristics spectrum.

Overall, our findings support an association between ADHD characteristics and negative risk-taking behavior, though our sample composition and nonclinical assessment of ADHD characteristics may limit its generalization. Our findings are in line with previous research showing that both children and adults with ADHD engage in more risk-taking, as evidenced by self-report measures, behavioral tasks, and virtual reality metrics (Pollak et al., 2019; Roberts et al., 2021). However, associations with positive and prosocial risk-taking are weaker and less consistent across analytical approaches. Furthermore, we confirm our expectation that the association between risk-taking and ADHD extends beyond internalizing symptoms such as depression, anxiety, and stress. Yet, the observed relationships cannot be attributed solely to ADHD traits, as they also appear to reflect broader mental health concerns. This is supported by the associations with internalizing symptoms, suggesting a shared influence. The interdependence may be explained by established links between risky behaviors and depression (e.g., Pozuelo et al., 2022), as well as the frequent co-occurrence of depressive symptoms in individuals with ADHD. Furthermore, the smaller effects observed between ADHD and positive or prosocial risk-taking may be explained by the fact that most expected positive consequences are future-oriented, and individuals with ADHD are known to be averse to delayed outcomes (Sonuga-Barke, 2005).

This pattern stands in contrast to the recent study by Braams et al. (2025), which examined clinically diagnosed adolescents with ADHD. Their findings revealed a higher likelihood of prosocial risk-taking in adolescents with ADHD compared to controls, but no significant differences in negative or positive risk-taking. The discrepancies between our findings and those reported by Braams et al. (2025) may be attributed to several factors. First, the relationship between risk-taking and ADHD may differ fundamentally between adolescents and adults. For example, the developmental peak in risky behavior typically observed during adolescence might be more pronounced in individuals with ADHD compared to controls, as discussed by Dekkers et al. (2022). Relatedly, the behavioral measures used to assess risk-taking in our study were originally designed for adolescent populations and may not fully reflect risk-taking tendencies in adults. This limitation is highlighted by our observation of an age-related decline in all forms of risk-taking from early to late adulthood, with older participants reporting a lower likelihood of engaging in such behaviors. This finding aligns with established evidence indicating that risk-taking peaks in late adolescence and gradually declines with age (Defoe et al., 2015; Duell et al., 2018, for a meta-analysis on age-related effects on risk-taking). Second, prosocial risk-taking may have been inadequately operationalized. This is reflected in the suboptimal internal consistency of the prosocial risk-taking scale in our study, a limitation also noted by Braams et al. (2025).

Further, in our data, all three forms of risk-taking were interrelated, suggesting that a higher reported likelihood of one type coincided with increased likelihoods of the others. Future research would benefit from a conceptual refinement and rigorous psychometric evaluation of prosocial risk-taking in adults, particularly in terms of its distinction from general positive and negative risk-taking. For example, previous studies testing the link between negative and positive risk taking in adolescents found that self-regulatory problems may lead to more negative but less positive risk-taking (Duell et al., 2019). A promising and psychometrically sound measure of prosocial risk-taking for adolescents was recently developed by van Rijn et al. (2025), providing a valuable foundation for the development of a similar measure in adults.

### Limitations and Future Directions

The present study should be interpreted in light of several limitations, resulting in future research directions. First, we adopted a dimensional approach to ADHD (Hudziak et al., 2007), examining correlational patterns within a non-clinical sample based on self-reported ADHD characteristics. It remains unclear whether these findings generalize to clinically diagnosed populations. Self-reported ADHD traits in community samples assess current experiences of ADHD like characteristics, but miss to assess retrospective childhood behavior and functional impairments typically observed in clinical cases (Gathje et al., 2008; Gordon et al., 2006). Although sensitivity of ADHD screenings are usually high, specificity is generally low resulting in low positive predictive value (Harrison & Edwards, 2023). Future studies should seek to replicate these findings in clinically referred samples.

Second, contrary to our expectations, our findings suggest that ADHD and internalizing symptoms jointly influence risk-taking behavior. This observation is noteworthy, as internalizing mental health conditions frequently co-occur with ADHD in clinical settings and may interact to shape different forms of risk-taking. Future research using clinical samples could benefit from advanced statistical methods, such as latent class analysis, to identify subgroups of individuals with ADHD and comorbid conditions who are particularly prone to engage in risky behavior.

Third, the measures of positive and prosocial risk-taking used in this study were originally developed for adolescents, and were adapted for their application on adults. However, to maximize utility in adult populations, future research should prioritize comprehensive item development and adaptation for adults, followed by factor-analytic validation to ensure the constructs are appropriately captured in adult clinical contexts. Thorough item analysis is relevant in order to arrive at psychometrically sound measures, which may improve low internal consistency of the prosocial risk-taking scale in the present study.

Fourth, our study assessed self-reported likelihoods of engaging in various risk-taking behaviors, which means hypothetical scenarios that may or may not accurately reflect actual behavior in real-life situations (e.g., as shown by Farnham et al., 2018; Szrek et al., 2012). To address this limitation, future research should incorporate behavioral measures, including observational studies of real-world risk-taking behaviors (e.g., participating in climate demonstrations during working time, or behaviors in real-world traffic). Real world risk-taking behavior may be more closely related with functional impairment how they are measured in clinical settings with self- and informant ratings.

Fifth, the correlational design of this study precludes any conclusions about causality. More controlled, ideally experimental studies are needed, that are conducted in applied settings, where participants are presented with real or simulated opportunities to engage in different types of risk-taking (e.g., real world or simulated cycling or car driving and behaviors at work or social settings).

Sixth, sample representativeness warrants consideration. The majority of participants in our final sample self-identified as female (56%), which may limit the generalizability of our findings to predominantly male populations. Furthermore, our approach to screening for ADHD characteristics resulted in 19% of the total sample being classified as having “elevated ADHD characteristics.” Given that self-reported ADHD screening tools are known to have high sensitivity (i.e., they capture most individuals with clinical ADHD) but low specificity (with positive predictive values around 26%; see Brevik et al., 2020, as reported in Harrison & Edwards, 2023), the proportion of positive screenings observed in our study falls within a realistic and plausible range compared with previous research employing similar methodologies and even the same screening instrument (Brevik et al., 2020).

Seventh, and finally, the mean item scores for negative, positive, and prosocial risk-taking hovered around the scale’s midpoint (i.e., scores of 3–4 on a 7-point scale), potentially indicating respondents’ uncertainty in evaluating their likelihood of engaging in the presented behaviors. Asking about actual (risky) behavior may show stronger and pronounced effects, and potentially differences between positive and negative risk-taking behavior, than asking about the likelihood of engaging in hypothetical behavior.

### Clinical Implications

Risk-taking refers to behaviors with variable and uncertain outcomes that may have either negative or positive consequences. While replication in clinically diagnosed adult ADHD samples using psychometrically validated measures is needed, our findings offer tentative support for the idea that ADHD characteristics may be linked not only to negative, but also to positive and prosocial forms of risk-taking. However, the relationship between ADHD and positive or prosocial risk-taking is likely more complex and may be influenced by a variety of factors, including internalizing conditions. These insights may contribute to a more nuanced understanding of ADHD, with potential implications for fostering positive self-concept, promoting acceptance of the condition, and supporting adaptive coping strategies (Schippers et al., 2022).

## Supplemental Material

sj-docx-1-jad-10.1177_10870547261432427 – Supplemental material for ADHD Characteristics Are Linked to Divergent Risk-Taking Behaviors Supplemental material, sj-docx-1-jad-10.1177_10870547261432427 for ADHD Characteristics Are Linked to Divergent Risk-Taking Behaviors by Anselm B. M. Fuermaier, Hui Dong, Yehuda Pollak, Barbara R. Braams and Tycho J. Dekkers in Journal of Attention Disorders

## Notes

1. To adopt a more neutral, nonmedical framing, we use the term “ADHD characteristics” rather than “ADHD symptoms” (for a commentary on this issue, see French et al., 2025)

## References

[bibr1-10870547261432427] Armstrong-Carter E. Do K. T. Moreira J. F. G. Prinstein M. J. Telzer E. H. (2021). Examining a new prosocial risk-taking scale in a longitudinal sample of ethnically diverse adolescents. Journal of Adolescence, 93, 222–233. 34826791 10.1016/j.adolescence.2021.11.002 PMC8688273

[bibr2-10870547261432427] Bangma D. F. Koerts J. Fuermaier A. B. M. Mette C. Zimmermann M. Toussaint A. K. Tucha L. Tucha O. (2019). Financial decision-making in adults with ADHD. Neuropsychology, 33(8), 1065–1077. 10.1037/neu0000571 31343233

[bibr3-10870547261432427] Barkley R. A. Cox D. J. (2007). A review of driving risks and impairments associated with attention deficit/hyperactivity disorder and the effects of stimulant medication on driving performance, Journal of Safety Research, 38(1), 113–128, 10.1016/j.jsr.2006.09.004 17303170

[bibr4-10870547261432427] Blais A. R. Weber E. U. (2006). A domain-specific risk-taking (DOSPERT) scale for adult populations. Judgment and Decision Making, 1, 33–47. 10.1017/S1930297500000334

[bibr5-10870547261432427] Braams B. R. van Rijn R. Leijser T. Dekkers T. J. (2025). The upside of ADHD-related risk-taking: Adolescents with ADHD report a higher likelihood of engaging in prosocial risk-taking behavior than typically developing adolescents. Journal of Attention Disorder, 21, 10870547251321882. 10.1177/10870547251321882 PMC12255846 39981819

[bibr6-10870547261432427] Brevik E. J. Lundervold A. J. Haavik J. Posserud M. B. (2020). Validity and accuracy of the adult attention-deficit/ hyperactivity disorder (ADHD) self-report scale (ASRS) and the Wender Utah Rating Scale (WURS) symptom checklists in discriminating between adults with and without ADHD. Brain and Behavior, 10, e01605. 10.1002/brb3.1605 PMC7303368 32285644

[bibr7-10870547261432427] Breyer J. L. Botzet A. M. Winters K. C. Stinchfield R. D. August G. Realmuto G. (2009). Young adult gambling behaviors and their relationship with the persistence of ADHD. Journal of Gambling Studies, 25, 227–238. 10.1007/s10899-009-9126-z 19283457 PMC2775442

[bibr8-10870547261432427] Charpentier C. J. Wu Q. Min S. Ding W. Cockburn J. O’Doherty J. P. (2024). Heterogeneity in strategy use during arbitration between experiential and observational learning. Nature Communications, 15(1), 4436. 10.1038/s41467-024-48548-y PMC11126711 38789415

[bibr9-10870547261432427] Cohen J. (2013). Statistical power analysis for the behavioral sciences. Academic Press.

[bibr10-10870547261432427] Cramér H. (1999). Mathematical methods of statistics (Vol. 26). Princeton University Press.

[bibr11-10870547261432427] Crone E. A. van Duijvenvoorde A. C. Peper J. S. (2016). Annual research review: Neural contributions to risk-taking in adolescence–developmental changes and individual differences. Journal of Child Psychology and Psychiatry, 57(3), 353–368. 26889896 10.1111/jcpp.12502

[bibr12-10870547261432427] Dayan H. Shoham R. Berger I. Khoury-Kassabri M. Pollak Y. (2023). Features of attention deficit/hyperactivity disorder and antisocial behaviour in a general population-based sample of adults. Criminal Behaviour & Mental Health, 33(3), 172–184. 10.1002/cbm.2288 37057691

[bibr13-10870547261432427] de Beurs E. Van Dyck R. Marquenie L. A. Lange A. Blonk R. W. B. (2001). De DASS: Een vragenlijst voor het meten van depressie, angst en stress [The DASS: A questionnaire for the measurement of depression, anxiety, and stress]. Gedragstherapie, 34(1), 35–53.

[bibr14-10870547261432427] Defoe I. N. Dubas J. S. Figner B. van Aken M. A. (2015). A meta-analysis on age differences in risky decision making: Adolescents versus children and adults. Psychological Bulletin, 141(1), 48–84. 10.1037/a0038088. 25365761

[bibr15-10870547261432427] Dekkers T. J. de Water E. Scheres A. (2022). Impulsive and risky decision-making in adolescents with attention-deficit/hyperactivity disorder (ADHD): The need for a developmental perspective. Current Opinion in Psychology, 44, 330–336. 10.1016/j.copsyc.2021.11.002 34953445

[bibr16-10870547261432427] Do K. T. Moreira J. F. G. Telzer E. H. (2017). But is helping you worth the risk? Defining prosocial risk-taking in adolescence. Developmental Cognitive Neuroscience, 25, 260–271. 28063823 10.1016/j.dcn.2016.11.008 PMC5461219

[bibr17-10870547261432427] Duell N. Steinberg L. (2019). Positive risk-taking in adolescence. Child Development Perspectives, 13(1), 48–52. 30774707 10.1111/cdep.12310 PMC6371981

[bibr18-10870547261432427] Duell N. Steinberg L. Icenogle G. Chein J. Chaudhary N. Di Giunta L. Dodge K. A. Fanti K. A. Lansford J. E. Oburu P. Pastorelli C. Skinner A. T. Sorbring E. Tapanya S. Uribe Tirado L. M. Alampay L. P. Al-Hassan S. M. Takash H. M. S.. Bacchini D. Chang L. (2018). Age patterns in risk taking across the world. Journal of Youth and Adolescence, 47(5), 1052–1072. 10.1007/s10964-017-0752-y 29047004 PMC5878702

[bibr19-10870547261432427] Dunn A. M. Heggestad E. D. Shanock L. R. Teilgard N. (2018). Intra-individual response variability as an indicator of insufficient effort responding: Comparison to other indicators and relationships with individual differences. The Journal of Business and Psychology, 33, 105–121. 10.1007/s10869-016-9479-0

[bibr20-10870547261432427] Farnham A. Ziegler S. Blanke U. Stone E. Hatz C. Puhan M. A. (2018). Does the DOSPERT scale predict risk-taking behaviour during travel? A study using smartphones. Journal of Travel Medicine, 25(1), tay064. 10.1093/jtm/tay064 30107438

[bibr21-10870547261432427] Flory K. Molina B. S. G. Pelham W. E. Jr. Gnagy E. Smith B. (2006). Childhood ADHD predicts risky sexual behavior in young adulthood. Journal of Clinical Child and Adolescent Psychology, 35(4), 571–577. 17007602 10.1207/s15374424jccp3504_8

[bibr22-10870547261432427] French B. Dekkers T. J. Barclay I. Black M. H. Bölte S. Daley D. Ernst J. Groom M. van Hulst B. M. de Jong M. Kaiser A. Kerner Auch Koener J. Kuntsi J. Michelini G. Price A. Purper-Ouakil D. Rijmen J. Wiersema J. R. , . . . Martin J. (2025). The power of words: Respectful language in ADHD research. The Lancet Psychiatry, 12(12), 876–879 40706601 10.1016/S2215-0366(25)00167-1

[bibr23-10870547261432427] Fuermaier A. B. M. Tucha L. Evans B. L. Koerts J. de Waard D. Brookhuis K. Aschenbrenner S. Thome J. Lange K. W. Tucha O. (2017). Driving and attention deficit hyperactivity disorder. Journal of Neural Transmission, 124(1), 55–67. 26419597 10.1007/s00702-015-1465-6 PMC5281661

[bibr24-10870547261432427] Gathje R. A. Lewandowski L. J. Gordon M. (2008). The role of impairment in the diagnosis of ADHD. Journal of Attention Disorders, 11(5), 529–537. 10.1177/1087054707314028 18259000

[bibr25-10870547261432427] Gordon M. Antshel K. Faraone S. Barkley R. Lewandowski L. Hudziak J. J. Biederman J. Cunningham C. (2006). Characteristics versus impairment: The case for respecting DSM-IV’s criterion D. Journal of Attention Disorders, 9(3), 465–475. 10.1177/1087054705283881 16481663

[bibr26-10870547261432427] Harrison A. G. Edwards M. J. (2023). The ability of self-report methods to accurately diagnose attention deficit hyperactivity disorder: A systematic review. Journal of Attention Disorders, 27(12), 1343–1359. 10.1177/10870547231177470 37366274

[bibr27-10870547261432427] Hershko S. Aronis A. Maeir A. Pollak Y. (2018). Dysfunctional eating patterns of adults with attention deficit hyperactivity disorder. The Journal of Nervous and Mental Disease, 206(11), 870–874. 30371641 10.1097/NMD.0000000000000894

[bibr28-10870547261432427] Ho D. Imai K. King G. Stuart E. Whitworth A. Greifer N . (2018). Package ‘matchit’. http://cran.uni-muenster.de/web/packages/MatchIt/MatchIt.pdf

[bibr29-10870547261432427] Holton G. A. (2004). Defining risk. Financial Analysts Journal, 60(6), 19–25.

[bibr30-10870547261432427] Huang J. L. Curran P. G. Keeney J. Poposki E. M. DeShon R. P . (2012). Detecting and deterring insufficient effort responding to surveys. Journal of Business and Psychology, 27(1), 99–114. 10.1007/s10869-011-9231-8

[bibr31-10870547261432427] Hudziak J. J. Achenbach T. M. Althoff R. R. Pine D. S. (2007). A dimensional approach to developmental psychopathology. International Journal of Methods in Psychiatric Research, 16(S1), S16–S23. 10.1002/mpr.217 PMC6879082 17623391

[bibr32-10870547261432427] Johnson J. A. (2005). Ascertaining the validity of individual protocols from web-based personality inventories. Journal of Research in Personality, 39(1), 103–129. 10.1016/j.jrp.2004.09.009

[bibr33-10870547261432427] Kaisari P. Dourish C. T. Higgs S. (2017). Attention deficit hyperactivity disorder (ADHD) and disordered eating behaviour: A systematic review and a framework for future research. Clinical Psychology Review, 53, 109–121. 28334570 10.1016/j.cpr.2017.03.002

[bibr34-10870547261432427] Kessler R. C. Adler L. Ames M. Demler O. Faraone S. Hiripi E. V. A. Walters E. E. (2005). The World Health Organization Adult ADHD Self-Report Scale (ASRS): A short screening scale for use in the general population. Psychological Medicine, 35(2), 245–256. 15841682 10.1017/s0033291704002892

[bibr35-10870547261432427] Kooij J. J. S. Boonstra A. M. Willemsen-Swinkels S. H. N. Bekker E. M. Noord I. Buitelaar J. K. (2008). Reliability, validity, and utility of instruments for self-report and informant report regarding characteristics of Attention-Deficit/Hyperactivity Disorder (ADHD) in adult patients. Journal of Attention Disorders, 11(4), 445–458. 18083961 10.1177/1087054707299367

[bibr36-10870547261432427] Kooij J. J. S. Buitelaar J. K. (1997). Zelfrapportage Vragenlijst over aandachtsproblemen en hyperactiviteit [Self-report Questionnaire for attentional problems and hyperactivity]: Not specified.

[bibr37-10870547261432427] Lovibond P. F. Lovibond S. H. (1995). The structure of negative emotional states: Comparison of the Depression Anxiety Stress Scales (DASS) with the Beck Depression and Anxiety Inventories. Behavioral Research and Therapy, 33(3), 335–343. 10.1016/0005-7967(94)00075 7726811

[bibr38-10870547261432427] Meade A. W. Craig S. B. (2012). Identifying careless responses in survey data. Psychological Methods, 17(3), 437–455. 10.1037/a0028085 22506584

[bibr39-10870547261432427] Mohamed S. M. Börger N. A. Geuze R. H. Van Der Meere J. J. (2015). Brain lateralization and self-reported characteristics of ADHD in a population sample of adults: A dimensional approach. Frontiers in Psychology, 6, Article 1418. 10.3389/fpsyg.2015.01418 PMC4585266 26441789

[bibr40-10870547261432427] Mohr-Jensen C. Muller Bisgaard C. Boldsen S. K. Steinhausen H.C (2019). Attention-Deficit/Hyperactivity disorder in childhood and adolescence and the risk of crime in young adulthood in a Danish nationwide study. Journal of the American Academy of Child and Adolescent Psychiatry, 58, 443–452. 30768385 10.1016/j.jaac.2018.11.016

[bibr41-10870547261432427] Molina B. S. Pelham W. E. (2014). Attention-deficit/hyperactivity disorder and risk of substance use disorder: Developmental considerations, potential pathways, and opportunities for research. Annual Review of Clinical Psychology, 10, 607–639. 10.1146/annurev-clinpsy-032813-153722 PMC4097844 24437435

[bibr42-10870547261432427] Ostergaard S. D. Dalsgaard S. Faraone S. V. Munk-Olsen T. Laursen T. M. (2017). Teenage parenthood and birth rates for individuals with and without attention-deficit/hyperactivity disorder: A nationwide cohort study. Journal of the American Academy of Child and Adolescent Psychiatry, 56(7), 578–584.e3. 10.1016/j.jaac.2017.05.003. 28647009

[bibr43-10870547261432427] Pollak Y. Dekkers T. J. Shoham R. Huizenga H. M. (2019). Risk-taking behavior in attention deficit/hyperactivity disorder (ADHD): A review of potential underlying mechanisms and of interventions. Current Psychiatry Reports, 21, 33. 10.1007/s11920-019-1019-y 30903380

[bibr44-10870547261432427] Pozuelo J. R. Desborough L. Stein A. Cipriani A (2022). Systematic review and meta-analysis: Depressive characteristics and risky behaviors among adolescents in low- and middle-income countries. Journal of the American Academy of Child and Adolescent Psychiatry, 61(2), 255–276. 10.1016/j.jaac.2021.05.005. 34015483

[bibr45-10870547261432427] Pratt T. C. Cullen F. T. Blevins K. R. Daigle L. Unnever J. D. (2002). The relationship of attention deficit hyperactivity disorder to crime and delinquency: A meta-analysis. International Journal of Police Science and Management, 4(4), 344–360.

[bibr46-10870547261432427] R Core Team. (2021). R: A language and environment for statistical computing. R Foundation for Statistical Computing (p. 2012).

[bibr47-10870547261432427] Roberts D. K. Alderson R. M. Betancourt J. L. Bullard C. C. (2021). Attention-deficit/hyperactivity disorder and risk-taking: A three-level meta-analytic review of behavioral, self-report, and virtual reality metrics. Clinical Psychology Review, 87, Article 102039. 10.1016/j.cpr.2021.102039. 34004385

[bibr48-10870547261432427] Romer D . (2010). Adolescent risk taking, impulsivity, and brain development: Implications for prevention. Developmental Psychobiology, 52(3), 263–276. 10.1002/dev.20442 20175097 PMC3445337

[bibr49-10870547261432427] Schippers L. M. Horstman L. I. Velde H. V. D. Pereira R. R. Zinkstok J. Mostert J. C. Hoogman M. (2022). A qualitative and quantitative study of self-reported positive characteristics of individuals with ADHD. Frontiers in Psychiatry, 13, Article 922788. 10.3389/fpsyt.2022.922788 PMC9597197 36311492

[bibr50-10870547261432427] Sibley M. H. (2021). Empirically-informed guidelines for first-time adult ADHD diagnosis. Journal of Clinical and Experimental Neuropsychology, 43(4), 340–351. 10.1080/13803395.2021.1923665 33949916

[bibr51-10870547261432427] Song Q. Zhang Y. Li X. Rudan I. (2021). The prevalence of adult attention-deficit/ hyperactivity disorder: A global systematic review and meta-analysis. Journal of Global Health, 11, Article 04009. 10.7189/jogh.11.04009 PMC7916320 33692893

[bibr52-10870547261432427] Sonuga-Barke E. J. (2005). Causal models of attention-deﬁcit/hyperactivity disorder: From common simple deﬁcits to multiple developmental pathways. Biological Psychiatry,57, 1231–1238. 15949993 10.1016/j.biopsych.2004.09.008

[bibr53-10870547261432427] Szrek H. Chao L.-W. Ramlagan S. Peltzer K. (2012). Predicting (un)healthy behavior: A comparison of risk-taking propensity measures. Judgement and Decision Making, 7(6), 716–727. 10.1017/S1930297500003260 24307919 PMC3846348

[bibr54-10870547261432427] Theule J. Hurl K. E. Cheung K. Ward M. Henrikson B. (2016). Exploring the relationships between problem gambling and ADHD: A meta analysis. Journal of Attention Disorder, 23(12), 1427–1437. 10.1177/1087054715626512 26832122

[bibr55-10870547261432427] van Rijn R. van Lange P. A. M. Krabbendam L. Braams B. R. (2025). Construction and validation of the prosocial adolescent risk-taking questionnaire (PAR-Q). Scientific Reports, 15(1), 21252. 10.1038/s41598-025-06034-5 PMC12214629 40594955

[bibr56-10870547261432427] Ward M. K. Meade A. W. (2022). Dealing with careless responding in survey data: Prevention, identification, and recommended best practices. Annual Review of Psychology, 74(1), 577–596. 10.1146/annurev-psych-040422-045007 35973734

[bibr57-10870547261432427] Wu Q. Hong Q. Kim N. Y. Adolphs R. Paul L. K. Charpentier C. J. (2025). Variability and stability of autistic traits in the general population: A systematic comparison between online and in-lab samples. Personality Neuroscience, 8, Article e5. 10.1017/pen.2025.10001 PMC12516608 41089295

[bibr58-10870547261432427] Wu Q. Oh S. Tadayonnejad R. Feusner J. D. Cockburn J. O’Doherty J. P. Charpentier C. J. (2024). Individual differences in autism-like traits are associated with reduced goal emulation in a computational model of observational learning. Nature Mental Health, 2, 1032–1044. 10.1038/s44220-024-00287-1 39734327 PMC11670734

[bibr59-10870547261432427] Yentes R. D. Wilhelm F. (2021). careless: Procedures for computing indices of careless responding. CRAN.

