# Supplementary material for: ADHD Characteristics Are Linked to Divergent Risk-Taking Behaviors
Source: J Atten Disord. 2026 Mar 31;30(8):1067–79. doi: 10.1177/10870547261432427 (PMC13328927; doi:10.1177/10870547261432427)
Supplement: sj-docx-1-jad-10.1177_10870547261432427 – Supplemental material for ADHD Characteristics Are Linked to Divergent Risk-Taking Behaviors [file sj-docx-1-jad-10.1177_10870547261432427.docx]

**Supplement**

**Risk taking items (Dutch)**

| **Vraag u bij elk van de volgende voorbeelden af: hoe waarschijnlijk is het dat ik dit zou doen?** | Risk taking type |
| --- | --- |
| 1. Toegeven dat uw smaak anders is dan die van uw vrienden. | Negative Risk-taking |
| 2. Kamperen in de vrije natuur, ver weg van de beschaving en de campings. | Negative Risk-taking |
| 3. Op een avond vijf of meer glazen alcohol drinken. | Negative Risk-taking |
| 4. Een aanzienlijk bedrag aan inkomsten niet opgeven in de belastingaangifte. | Negative Risk-taking |
| 5. Ten aanzien van een belangrijk thema van mening verschillen met een autoriteit. | Negative Risk-taking |
| 6. Bij het pokeren een daginkomen inzetten. | Negative Risk-taking |
| 7. Een affaire hebben met een getrouwde man of vrouw. | Negative Risk-taking |
| 8. Het werk van iemand anders als het uwe presenteren. | Negative Risk-taking |
| 9. 5% van uw jaarinkomen in zeer speculatieve aandelen investeren. | Negative Risk-taking |
| 10. De inkomsten van een dag inzetten op de uitkomst van een sportwedstrijd (bijv. voetbal of basketbal). | Negative Risk-taking |
| 11. Aan onbeschermde sex doen. | Negative Risk-taking |
| 12. Rijden zonder veiligheidsgordel. | Negative Risk-taking |
| 13. Een cursus parachutespringen volgen. | Negative Risk-taking |
| 14. Een baan waar u echt van geniet verkiezen boven een baan met veel zekerheid. | Negative Risk-taking |
| 15. Een gevoelige kwestie waar u in gelooft tijdens een werkvergadering verdedigen. | Negative Risk-taking |
| 16. In de zon gaan liggen zonder uzelf met zonnebrandcrème te hebben ingesmeerd. | Negative Risk-taking |
| 17. 's Nachts alleen door een onveilig stadsdeel naar huis gaan. | Negative Risk-taking |
| 18. Verhuizen naar een stad ver van uw familie. | Negative Risk-taking |
| 19. Halverwege de dertig van loopbaan veranderen. | Negative Risk-taking |
| 20. Uw kleine kinderen alleen thuislaten terwijl u iets gaat kopen. | Negative Risk-taking |
| 21. Op een blind date gaan. | Positive Risk-taking |
| 22. Iemand verdedigen die in een trein op een ongepaste manier wordt benaderd. | Prosocial Risk-taking |
| 23. Een vriend of vriendin vertellen dat zijn of haar partner vreemdgegaan is. | Prosocial Risk-taking |
| 24. Geld lenen aan een vriend of vriendin terwijl u vermoedt dat u het geld niet terug zou kunnen krijgen. | Prosocial Risk-taking |
| 25. Aan een cursus over een onbekend onderwerp deelnemen. | Positive Risk-taking |
| 26. Uw leidinggevende erop wijzen dat er etensresten tussen zijn of haar tanden hangen. | Prosocial Risk-taking |
| 27. Alleen op reis gaan (waar u nog niet eerder bent geweest). | Positive Risk-taking |
| 28. Een toespraak houden voor veel mensen . | Positive Risk-taking |
| 29. Op een feestje een onbekend persoon aanspreken. | Positive Risk-taking |
| 30. Wegblijven van het werk om deel te nemen aan een klimaatprotest. | Prosocial Risk-taking |
| 31. Voor een collega inspringen door op het werk namens hem of haar te tekenen. | Prosocial Risk-taking |
| 32. Een hond of kat van een drukke weg redden. | Prosocial Risk-taking |
| 33. Uzelf een nieuwe look aanmeten (bijv. kapsel, outfit) zonder te weten hoe anderen erop zullen reageren. | Positive Risk-taking |
| 34. Deelnemen aan een sportactiviteit die u nog nooit eerder hebt gedaan waarbij u zichzelf mogelijk voor schut zou kunnen zetten. | Positive Risk-taking |
| 35. In een restaurant of tijdens een diner iets onbekends nemen dat u misschien niet lekker vindt. | Positive Risk-taking |
| 36. Voor een opname bij een band of theatergroep iets zingen of voordragen. | Positive Risk-taking |
| 37. Iemand die u mag, maar die niet populair is bij anderen, uitnodigen voor een feestje | Prosocial Risk-taking |
| 38. Iemand verdedigen die gepest wordt. | Prosocial Risk-taking |
| 39. Een uitnodiging voor een verjaardag accepteren waarbij u de gasten niet kent. | Positive Risk-taking |
| 40. Een collega vertellen dat er slecht over hem of haar wordt gesproken. | Prosocial Risk-taking |

**Risk taking items (English)**

| **For each of the following examples, ask yourself: How likely am I to do this?** | **Risk taking type** |
| --- | --- |
| 1. Admit that your taste is different from that of your friends. | Negative Risk-taking |
| 2. Camping in the great outdoors, far away from civilisation and campsites. | Negative Risk-taking |
| 3. Drinking five or more alcoholic drinks in one evening. | Negative Risk-taking |
| 4. Failure to declare a significant amount of income on your tax return. | Negative Risk-taking |
| 5. To disagree with an authority on an important issue. | Negative Risk-taking |
| 6. Betting a day's income when playing poker. | Negative Risk-taking |
| 7. Having an affair with a married man or woman. | Negative Risk-taking |
| 8. Presenting someone else's work as your own. | Negative Risk-taking |
| 9. Invest 5% of your annual income in highly speculative shares. | Negative Risk-taking |
| 10. The income from betting on the outcome of a sporting event (e.g. football or basketball) for one day. | Negative Risk-taking |
| 11. Engaging in unprotected sex. | Negative Risk-taking |
| 12. Driving without a seatbelt. | Negative Risk-taking |
| 13. Take a course in parachuting. | Negative Risk-taking |
| 14. Choosing a job you really enjoy over a job with a lot of security. | Negative Risk-taking |
| 15. Defending a sensitive issue you believe in during a work meeting. | Negative Risk-taking |
| 16. Lying in the sun without applying sunscreen. | Negative Risk-taking |
| 17. Walking home alone at night through an unsafe part of town. | Negative Risk-taking |
| 18. Moving to a city far away from your family. | Negative Risk-taking |
| 19. Changing careers in your mid-thirties. | Negative Risk-taking |
| 20. Leaving your young children home alone while you go shopping. | Negative Risk-taking |
| 21. Going on a blind date. | Positive Risk-taking |
| 22. Defending someone who is approached inappropriately on a train. | Prosocial Risk-taking |
| 23. Telling a friend that his or her partner has been unfaithful. | Prosocial Risk-taking |
| 24. Lending money to a friend when you suspect that you may not get it back. | Prosocial Risk-taking |
| 25. Taking a course on an unfamiliar subject. | Positive Risk-taking |
| 26. Pointing out to your manager that there is food stuck between his or her teeth. | Prosocial Risk-taking |
| 27. Travelling alone (to a place you have not been before). | Positive Risk-taking |
| 28. Giving a speech in front of many people. | Positive Risk-taking |
| 29. Approaching a stranger at a party. | Positive Risk-taking |
| 30. Staying away from work to participate in a climate protest. | Prosocial Risk-taking |
| 31. Filling in for a colleague by signing on their behalf at work. | Prosocial Risk-taking |
| 32. Rescuing a dog or cat from a busy road. | Prosocial Risk-taking |
| 33. Giving yourself a new look (e.g. hairstyle, outfit) without knowing how others will react. | Positive Risk-taking |
| 34. Participating in a sporting activity that you have never done before, where you could potentially embarrass yourself. | Positive Risk-taking |
| 35. Ordering something unfamiliar in a restaurant or during a dinner that you may not like. | Positive Risk-taking |
| 36. Sing or recite something for an audition with a band or theatre group. | Positive Risk-taking |
| 37. Inviting someone you like, but who is not popular with others, to a party. | Prosocial Risk-taking |
| 38. Defending someone who is being bullied. | Prosocial Risk-taking |
| 39. Accepting an invitation to a birthday party where you do not know the guests. | Positive Risk-taking |
| 40. Telling a colleague that people are speaking badly about him or her. | Prosocial Risk-taking |

Table S1. *Group characteristics and risk-taking behavior by ADHD characteristics and age*

| Characteristic | Elevated ADHD characteristics (young, *n* = 55) ^a^ | Comparison group (young, *n* = 59) ^b^ | *p*-value | Cohen’s *d*/  Cramér’s *V ^c^* | Elevated ADHD characteristics  (old, *n* = 59) ^a^ | Comparison group (old, *n* = 55) ^b^ | *p*-value | Cohen’s *d*/  Cramér’s *V ^c^* |
| --- | --- | --- | --- | --- | --- | --- | --- | --- |
| Gender identity (Female/Male/Non-binary) ^d^ | 38/16/1 | 43/16/0 | 0.93 | 0.30 | 24/34/1 | 24/31/0 | 0.96 | 0.02 |
| Age (in years) | 22.91 ± 3.71 | 22.68 ± 3.72 | 0.72 | 0.06 | 45.97 ± 11.03 | 46.58 ± 11.12 | 0.76 | 0.06 |
| %Education (1/2/3/4/5/6/7) ^e^ | 0/0/17/15/17/24/27 | 0/0/7/14/21/28/30 | 0.69 ^f^ | 0.16 ^f^ | 0/0/20/2.4/2.4/49/27 | 2.1/2.1/23/13/2.1/50/8.3 | - | - |
| Risk taking behavior | | | | | | | | |
| -Negative risk taking | 68.89 ± 13.51 | 63.68 ± 13.27 | 0.04* | 0.39 | 68.69 ± 12.57 | 60.98 ± 12.78 | 0.01** | 0.60 |
| -Positive risk taking | 41.56 ± 9.54 | 40.58 ± 10.76 | 0.51 | 0.10 | 42.02 ± 10.80 | 38.85 ± 11.03 | 0.20 | 0.29 |
| -Prosocial risk taking | 43.75 ± 6.24 | 43.27 ± 8.45 | 0.74 | 0.06 | 45.03 ± 6.66 | 42.62 ± 7.23 | 0.10 | 0.35 |
| Symptom scores | | | | | | | | |
| -ADHD | 34.80 ± 7.86 | 14.90 ± 6.71 | <.001*** | 2.71 | 32.63 ± 7.67 | 10.93 ± 5.54 | <.001*** | 3.20 |
| -Depression | 13.78 ± 4.47 | 9.31 ± 2.50 | <.001*** | 1.24 | 15.07 ± 4.95 | 9.31 ± 3.59 | <.001*** | 1.32 |
| -Anxiety | 14.07 ± 4.09 | 9.59 ± 2.50 | <.001*** | 1.32 | 13.12 ± 3.87 | 8.89 ± 2.63 | <.001*** | 1.26 |
| -Stress | 16.25 ± 4.25 | 10.36 ± 2.60 | <.001*** | 1.68 | 17.08 ± 3.87 | 9.76 ± 2.91 | <.001*** | 2.11 |

*Note*

^a^ Elevated ADHD characteristics was operationally defined as endorsing ≥ 5 symptoms of inattention and/or hyperactivity/impulsivity. Groups were split by median age (30.5 years) into young and old adults.

^b^ Comparison group was sampled from participants who did not meet the criteria for elevated ADHD characteristics

^c^ Cohen’s *d* and Cramér’s *V* values refer to the comparison between the elevated ADHD characteristics group and the comparison group within the same age group.

^d^  Non-binary participants were not considered in the group comparison.

^e^ Education (1/2/3/4/5/6/7) = Ordinal presentation of Dutch education level, ranging from primary school without educational training until scientific education in BSc/MSc. Some categories have small sample sizes; *p* values for categorical comparisons should be interpreted cautiously.

^f^ p-values and Cramér’s V for Education were calculated on the all sample (young + old) using Fisher’s exact test due to small cell counts in subgroup analyses.

Significant differences between groups are indicated by **p*<.05, ***p*<.01, and ****p*<.001.
